# Genotypic variation in the response of soybean to elevated CO_2_

**DOI:** 10.1002/pei3.10065

**Published:** 2021-12-08

**Authors:** José C. Soares, Lars Zimmermann, Nicolas Zendonadi dos Santos, Onno Muller, Manuela Pintado, Marta W. Vasconcelos

**Affiliations:** ^1^ CBQF—Centro de Biotecnologia e Química Fina—Laboratório Associado Escola Superior de Biotecnologia Universidade Católica Portuguesa Porto Portugal; ^2^ Field Lab Campus Klein Altendorf University of Bonn Rheinbach Germany; ^3^ Institute for Bio‐ and Geosciences IBG‐2: Plant Sciences Forschungszentrum Jülich GmbH Jülich Germany

**Keywords:** elevated CO_2_, genetic variation, grain quality, minerals, photosynthesis, soybean

## Abstract

The impact of elevated CO_2_ (eCO_2_) on soybean productivity is essential to the global food supply because it is the world's leading source of vegetable proteins. This study aimed to understand the yield responses and nutritional impact under free‐air CO_2_ enrichment (FACE) conditions of soybean genotypes. Here we report that grain yield increased by 46.9% and no reduction in harvest index was observed among soybean genotypes. Elevated CO_2_ improved the photosynthetic carbon assimilation rate, leaf area, plant height, and aboveground biomass at vegetative and pod filling stages. Besides the positive effects on yield parameters, eCO_2_ differentially affected the overall grain quality. The levels of calcium (Ca), phosphorous (P), potassium (K), magnesium (Mg), manganese (Mn), iron (Fe), boron (B), and zinc (Zn) grain minerals decreased by 22.9, 9.0, 4.9, 10.1, 21.3, 28.1, 18.5, and 25.9% under eCO_2_ conditions, respectively. Soluble sugars and starch increased by 9.1 and 16.0%, respectively, phytic acid accumulation increased by 8.1%, but grain protein content significantly decreased by 5.6% across soybean genotypes. Furthermore, the antioxidant activity decreased by 36.9%, but the total phenolic content was not affected by eCO_2_ conditions. Genotypes, such as Winsconsin Black, Primorskaja, and L‐117, were considered the most responsive to eCO_2_ in terms of yield enhancement and less affected in the nutritional quality. Our results confirm the existence of genetic variability in soybean responses to eCO_2_, and differences between genotypes in yield improvement and decreased sensitivity to eCO_2_ in terms of grain quality loss could be included in future soybean selection to enable adaptation to climate change.

## Graphical abstract

‐ Intraspecific variation among soybean genotypes affect the responses under eCO2conditions.‐ eCO2 significantly increased soybean yield.‐ Grain quality was affected by CO2 enrichment.

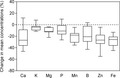

## 1 INTRODUCTION

In the 20 million years preceding the industrial revolution, atmospheric CO_2_ concentration in the atmosphere was below 280 ppm but continued to increase since then and reached almost 410 ppm (http://www.esrl.noaa.gov/gmd/ccgg/trends/) by 2020. In the future, with current trends, it will probably exceed 550 ppm by 2050 (IPCC, 2014). Therefore, plants are facing unprecedented levels of CO_2_ concentration, and it is highly questionable that they could adapt to this change so quickly (Bishop et al., 2015). Several changes in terms of growth, physiology, biochemical, and genetic traits takes place in plants exposed to elevated CO_2_ (eCO_2_) conditions (Palit et al., 2020). Thus, eCO_2_ has been reported to stimulate plant growth, and photosynthesis of several crops, and to reduce stomatal conductance (*g*_s_), leading to a greater transpiration efficiency (Asif et al., 2018; Bourgault et al., 2017; Hajiboland, 2012; Palit et al., 2020; Singh et al., 2016; Soares et al., 2019). The extent of the response varies between and within species, experimental conditions (Ainsworth & Long, 2005), and according to the interactions with climate changes and soil conditions (Bishop et al., 2015). The effects of eCO_2_ could also mitigate the damaging effects on yield due to other aspects of climate change such as rising temperature, increased frequency and intensity of droughts, and increased contact of vegetation to atmospheric water vapor pressure deficit (Abdelgawad et al., 2015; Bencke‐Malato et al., 2019; Bishop et al., 2015).

Soybean is an important crop consumed globally and the most extensively grown legume worldwide providing an essential source of protein and oil (Ainsworth et al., 2012; Kumar & Pandey, 2020). Nowadays, there is a growing demand for the consumption of legumes due to their high protein content, low in calories and glycemic index, and because they provide various health benefits (Kumar & Pandey, 2020). Global soybean production has steadily increased growing its production from 161 to 348 million tons in the last two decades (http://www.fao.org/faostat/en/#data), due to improved agronomy techniques and selection of cultivars suited to a wide range of environments (Ainsworth et al., 2012). CO_2_‐based responses in soybean have been extensively investigated, either in controlled and field experiments (Ainsworth et al., 2002; Kimball, 2016; Leakey et al., 2009). However, under FACE conditions, most studies have been carried out with one or a few genotypes (Bunce, 2014, 2016; Hao et al., 2014, 2016; Rosenthal et al., 2014), and to gain more knowledge about the adaption process to eCO_2_, it is essential to consider the intraspecific variability in yield responses. Bishop et al. (2015) investigated the intraspecific variation in the response of 18 soybean genotypes to increased CO_2_ (550 ppm) under FACE conditions. On average, there was an increase in biomass by 22%, and seed yield by 9%, partially because most genotypes showed a reduction in the partitioning of energy to seeds. In a controlled environment, Ziska et al. (2001) investigated nine soybean genotypes, and observed significant differences in the magnitude of the yield response under eCO_2_ conditions (710 ppm). Similar findings were observed by Soares et al. (2019) studying 17 soybean genotypes in a controlled environment, and the range of yield responses to eCO_2_ (800 ppm) was −23.8% to 39.6%. Considering the effects of eCO_2_ on soybean grain quality, results from a previous study suggest that eCO_2_ decreased soybean grain protein in open‐top chambers (Li et al., 2018). Myers et al. (2014) also found that eCO_2_ was associated with reduced protein content in C3 grasses, wheat, and rice grains, and with a small decrease in field pea although there was no significant effect in soybean under FACE conditions. Besides, the concentration of several minerals are significantly influenced by eCO_2_ which could affect the human nutrition in the upcoming future (Köhler et al., 2018). Using a meta‐analysis, Loladze (2014) showed that eCO_2_ declines the overall mineral concentrations by 8% in a range of C3 plants, reflecting foliar and edible tissues, using FACE and non‐FACE studies. It was also reported that C3 grains and legumes have reduced content of zinc (Zn) and iron (Fe) under FACE conditions (Myers et al., 2014). In another study, exposure to eCO_2_ during consecutive seasons decreased nitrogen (N), potassium (K), calcium (Ca), protein, and total amino acid concentrations in wheat grains, even though the starch concentration was not significantly affected (Li et al., 2019). Moreover, it is also important to consider phytate which is a phosphate storage molecule present in most plants, and a strong inhibitor of Fe, Zn, and Ca absorption (Gibson et al., 2010). Myers et al. (2014) measured phytate in plants grown under eCO_2_ and observed a significant reduction in wheat, but there was no decrease in phytate concentration in rice, field peas, soybeans, maize, and sorghum. Still, the combined analysis of minerals and phytate could provide a more thorough understanding on the impact of eCO_2_ on mineral bioavailability. There is even less information about the responses to eCO_2_ in terms of sugar concentration and on the antioxidant capacity in the grain of legumes. Dong et al. (2018) conducted a meta‐analysis suggesting that eCO_2_ increases the concentration of total soluble sugar, total antioxidant capacity, total phenols, total flavonoids, and ascorbic acid in the edible part of vegetables. In contrast, Zheng et al. (2020) proposed that the content of soluble sugars in soybean grains was not affected by eCO_2_, but the levels of natural antioxidants decreased. In another study, conducted using open‐top chambers, the total phenolic content (TPC) of two rice varieties decreased at eCO_2_ (Goufo et al., 2014). Therefore, most studies looking at the effects of eCO_2_ have focused on either the physiological or the nutritional responses, and very few have combined these two components to explain the basis for the impacts of eCO_2_ on nutrient accumulation. We therefore hypothesized that genetic selection toward CO_2_‐based responses for yield and grain quality is likely to involve a range of characteristics that balance sink and source associations. In this study, we analyzed the genotypic variation in soybean yield responses under field conditions. At the same time, we assessed leaf photosynthesis parameters, and grain quality, specifically, protein concentration, minerals, sugar, starch, phytic acid, phenolics content, and antioxidant activity.

## 2 MATERIAL AND METHODS

### 2.1 Research site and experimental design

This study was conducted at the FACE facility from the experimental station of the University of Bonn located at Campus Klein‐Altendorf (50°37'30.5"N 6°59'15.8"E, 160 m above sea level) in Germany. The soil is a loamy‐clay silt soil (luvisol) with a pH of 6.6 (1:5 soil:water), organic carbon of 1.84%, and a total N of 1.07 g/kg. During the growing season in 2018, the average precipitation and daytime temperature in June, July, August, and September was 44.7, 29.4, 19.1, and 37.1 mm and 17.8, 21.0, 19.8, 14.9 °C, respectively. The soil was not irrigated or fertilized, only receiving water through rainfall. Soybeans were planted on 30 May 2018. The FACE facility, consisted of two blocks, each containing two 17.5 m diameter octagonal plots. The CO_2_ concentration at the center of the ring was frequently monitored, and CO_2_ was released from the peripheral emission tubes at 0.5 m above the canopy. The emission source was chosen based on the current wind direction to maintain CO_2_ concentration within the ring at a level of 200 ppm above that in the ambient CO_2_ (aCO_2_) plots. The experimental design was a split‐pot model design (main plot = CO_2_ and split‐pot = genotypes) with two replicates. Within each block, one plot was at current CO_2_ concentration of 400 ppm, and one plot was fumigated with CO_2_ to 600 ppm using the FACE system. Each plot was divided into 52 of 1.5 m × 3 m subplots, and plants were sown in rows with 0.45 m spacing at a sowing density of 20 plants/m^2^. One side of the ring was subdivided into 26 subplots and planted with common bean, and the other side was planted with a range of soybean genotypes described in Table 1 and used in the current study. Each genotype occupied the same position in each ring and was randomly replicated in two subplots of each ring. Plots were fumigated with eCO_2_ during daylight from emergence to maturity using the FACE system.

**TABLE 1:** Description and ranks of yield response to eCO_2_ in soybean genotypes grown in growth chamber (Soares, Deuchande, et al., 2019), or in FACE plots, where 1 is the rank of the most responsive and 13 is the least responsive

Acession no	GH	Common name	Origin	Growth chamber	Yield stimulation	FACE	Yield stimulation	Average rank
PI 437101	I	DV‐0197^a^	Russia	9	−	13	−	11
PI 417554	I	EM^a^	Poland	3	+	12	−	7.5
PI 437413	I	Ussurijscaja^a^	Russia	11	−	11	−	11
PI 361097 A	I	Novosadska^a^	Serbia	4	−	10	+	7
PI 319537 A	I	Tono^a^	China	8	−	9	+	8.5
PI 538409	D	Shironomai^a^	Japan	2	+	8	+	5
PI 319534 A	I	Honshu^a^	China	6	−	7	+	6.5
PI 445829 A	I	Dunayka^a^	Romania	5	−	6	+	5.5
PI 153271	I	WB^a^	Belgium	1	+	4	+	2.5
PI 361085 A	I	L‐117^a^	Romania	12	−	5	+	8.5
PI153245	I	VDGY^a^	Germany	nd	nd	2	+	−
PI 437224	I	Cschi675^a^	Moldova	7	−	3	+	5
PI 378676 A	I	Primorskaja^a^	Russia	10	−	1	+	5.5

Abbreviations: D, determinate; EM, Early Mandarin; GH, growth habit; I, indeterminate; VDGY, Van Dieckman Green‐Yellow; WB, Wisconsin Black. (+) significant grain yield stimulation; (−) no significant grain yield stimulation; (nd) not determined.

^a^ Obtained from USDA‐ARS via Germplasm Resources Information Network (Washington, USA)

### 2.2 Crop growth and yield

All soybean genotypes, but one (VDGY), were previously grown in a growth chamber experiment (Soares, Deuchande, et al., 2019). Sampling points were determined at vegetative (V3–V4), and pod filling (R4) stages (Fehr et al., 1971). Three plants from each subplot were harvested for determination of leaf area (LI‐3100C area meter, LI‐COR), plant height, and aboveground dry weight after drying to constant weight at 60ºC in a forced‐air oven. Moreover, Soil and Plant Analyzer Development (SPAD) readings were conducted with a portable chlorophyll meter (Konica Minolta SPAD‐502 Plus; Minolta), using the first expanded trifoliate leaf from three plants. At maturity (R8), 10 plants from each subplot were taken to assess the number of pods per plant, the number of seeds per pod, number of seeds per plant, the average mass of 100 seeds, harvest index, and grain yield.

### 2.3 Gas exchange measurements

Gas exchange parameters were performed from each subplot in the last fully expanded leaves of three plants, at vegetative and pod filling stages. Rates of photosynthesis were determined between 10 and 16 h on clear sunny days. Leaf photosynthetic carbon assimilation rate (*A*_sat_), transpiration rate (*T*_r_), and *g*_s_ were measured with a portable gas exchange system incorporating an infrared CO_2_ and water vapor analyzers (LI‐COR 6400, LI‐COR). The CO_2_ concentration in the leaf chamber was controlled by the LI‐COR CO_2_ injection system, and irradiance of 1500 µmol photons/(m^2^ s) supplied by a built‐in LED lamp (red/blue). The temperature in the leaf chamber configured to 25ºC, and CO_2_ concentration to 400 or 600 ppm for each treatment. Instantaneous water‐use efficiency was calculated as *A*_sat_/*g*_s_.

### 2.4 Light‐induced fluorescence transient (LIFT) device

The LIFT method is a distinctive approach to probe photosystem II from a distance under natural conditions (Muller et al., 2018). The LIFT instrument (Version LIFT‐REM, Soliense Inc.) was equipped with a blue light‐emitting diode (LED) (445 nm), a STS‐VIS spectrometer (Ocean Optics), and two RGB cameras (FLIR Integrated Imaging Solutions Inc.). Subsaturating actinic LED flashlets in fast repetition rate (FRR) induce the maximum fluorescence yield and monitor its relaxation with decreasing repetition rates. Chlorophyll fluorescence is detected at 685 (±10) nm. The FRR flash was used with an excitation phase of 0.75 ms consisting of 300 flashlets. The relaxation phase included 127 flashlets triggered at decreasing repetition rate and lasted for 200 ms. Fluorescence measurements were performed in the last fully expanded leaves of three plants from each subplot with five measurements per plant at vegetative and pod filling stages. The LIFT instrument was fitted to a phenotyping bike with a track width of 3 m allowing top canopy measurements from 60 to 80 cm. The operational procedures of the system were described in a previous experiment (Keller et al., 2019).

### 2.5 Grain nutritional analysis

Ten seeds from independent plants at each subplot were pooled together and used for subsequent nutritional analysis. The mean values for each plot were treated as one replicate.

#### 2.5.1 Mineral analysis

Grain mineral analysis was performed as reported by Soares, Deuchande, et al. (2019). The seed material (200 mg) was mixed with 5 ml of HNO_3_ 65% (v/v), and 1 ml of H_2_O_2_ 30% (v/v) in a Teflon reaction vessel and heated in a SpeedwaveTM MWS‐3+ microwave system. Digestion procedure was achieved as follows: 130°C/10 min, 160°C/15 min, 170°C/12 min, 100°C/7 min, and 100°C/3 min. Each solution of the digestion procedure was brought to 50 ml with ultrapure water, and determination of mineral concentrations performed using the ICP‐OES Optima 7000 DV. The assays were performed in duplicates and mean values calculated.

#### 2.5.2 Determination of protein concentration

For each sample, a total of 75 mg of flour was collected and analyzed for protein concentration (*N* × 5.5) using a Leco N analyzer (Model FP‐528, Leco Corporation). The assays were performed in duplicates and mean values calculated.

#### 2.5.3 Phytic acid determination

The colorimetric Wade reagent method was used for detecting phytic acid as described by Gao et al. (2007) with some adjustments. A total of 50 mg of flour was mixed with 1 ml of 0.8 N HCl:10% Na_2_SO_4_, shaken at 220 rpm during 16–24 h, and centrifuged at 3000 g for 20 min at 10ºC. The extract was stored at 4ºC in the dark for further analysis. Then, 30 µl of extract was mixed with 720 µl of distilled water and 250 µl of Wade's Reagent, vortexed for 10 s, and an aliquot (200 µl) was read at 540 nm using a microplate reader (Synergy H1). The assays were performed in duplicates and mean values calculated.

#### 2.5.4 Determination of total sugars and starch

The sugar extraction was determined based on the protocol of Chow and Landhausser (2004). For each sample, 100 mg was extracted three times with 5 ml of 80% ethanol (v/v), by boiling the samples in a 95ºC water bath for 10 min. After each extraction, the tubes were centrifuged at 3000 rpm for 5 min, and supernatants combined for sugar analysis. Sugar quantification followed the microplate phenol–sulfuric acid assay developed by Masuko et al. (2005). Total starch was determined with kit from Megazyme according to AOAC method 996.11 (AOAC, 2006). The assays were performed in duplicates and mean values calculated.

#### 2.5.5 Extraction of phenolic compounds

For the preparation of the phenolic extract, 500 mg of each sample was mixed with 10 ml of acetone/water/acetic acid (70:29.5:0.5, v/v/v), and the extract was shaken overnight at 300 rpm in the dark using an orbital shaker (Zhou et al., 2017). Then, the extract was centrifuged at 1600 rpm for 10 min, and the supernatant stored at 4°C in the dark until further use.

#### 2.5.6 Total Phenolic Content

The TPC assay was performed using the Folin–Ciocalteu colorimetric method as described by Ramos et al. (2019), with slight variations. In a 96‐well plate, 150 µl of Folin–Ciocalteu reagent, and 75 µl of sodium carbonate solution (75 g/L) were added to 30 µl of soybean extracts. The mixture was incubated at room temperature in the dark and the absorbance was measured after 60 min at 750 nm, in a Thermo Scientific MultiskanTM FC microplate reader (Thermo Fisher Scientific Inc.). TPC in each sample was determined using a standard curve prepared by gallic acid (0.025–0.5 mg/ml). The result was expressed as mg of gallic acid equivalent per gram (mg GAE/g) of soybean. The assays were performed in duplicates and mean values calculated.

#### 2.5.7 Antioxidant Activity—ABTS radical cation scavenging effect

The phenolic extract was used for measuring the antioxidant activity by the ABTS radical scavenging assay according to Goncalves et al. (2009). Daily, the concentration of ABTS working solution was adjusted to an initial absorbance of 0.7 at 734 nm. Then, in a 96‐well plate, 280 µl of ABTS solution was added to 20 µl of sample or Trolox or solvent. After that, the mixture was allowed to react for 5 min in the dark, and the absorbance was immediately recorded at 734 nm, using a Thermo Scientific MultiskanTM FC microplate reader (Thermo Fisher Scientific Inc.). Trolox was used as the reference antioxidant, and the result was expressed as mmol of Trolox equivalent per gram (mmol TE/g) of soybean. The assays were performed in duplicates and mean values calculated.

### 2.6 Statistical analysis

The 13‐genotype experiment was analyzed with a split‐plot mixed model analysis of variance, where CO_2_ was treated as the main factor, and genotype as the split factor, using the general linear model procedure of SPSS (28.0 SPSS Inc.). Where significant differences were found, means were compared using Tukey's Test at 0.05 significance level. For some dependent variables, the variance was heterogeneous and, so a transformation was performed before the statistical analysis. The correlations among seed yield and agronomic traits were performed using Pearson's product‐moment correlation (*r*) at 0.05 significance level. Thus, mean response of each of the genotypes exposed to eCO_2_ was used to investigate how seed yield response to eCO_2_ (eCO_2_/aCO_2_) correlated with different yield parameters. Principal component analysis (PCA) was performed on grain nutritional analysis and yield data using PAST 4 (Paleontological statistics software package for education and data analysis, version 4.03.

## 3 RESULTS

### 3.1 Yield responses to eCO_2_

**FIGURE 1:**
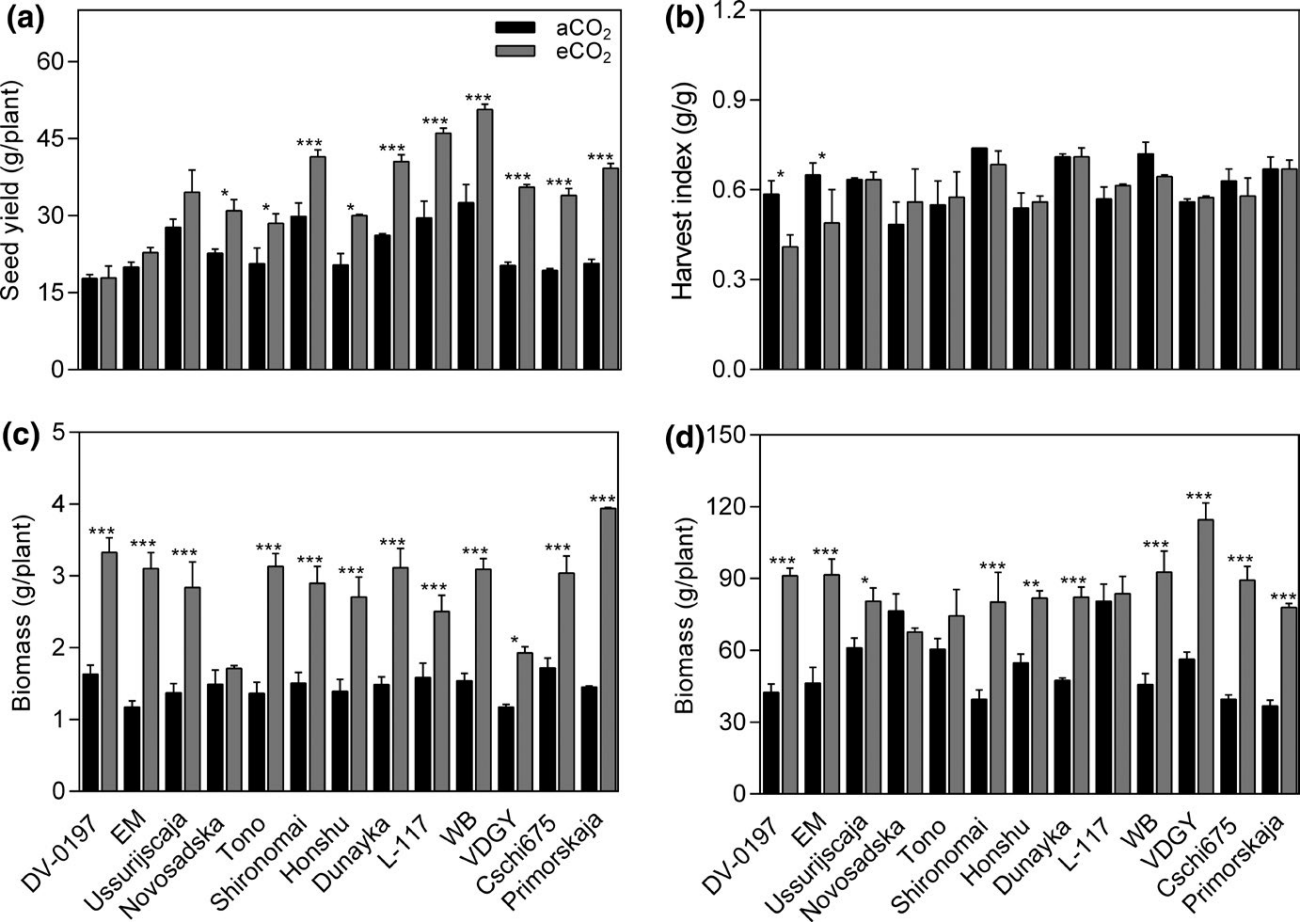
Genotypic variation in (a) soybean seed yield, (b) harvest index, and aboveground biomass at (c) vegetative and (d) pod filling stages under eCO_2_. Bars show the mean value of each variable ± standard error in 13 genotypes grown at the FACE facility in 2018. Bars with asterisk(s) indicate significant effects of CO_2_ for each genotype tested. Ten plants from each subplot were sampled to assess the grain yield and harvest index, and three plants from each subplot were sampled to assess the aboveground biomass. * *p* < .05; ** *p* < .001; *** *p* < .0001

**FIGURE 2:**
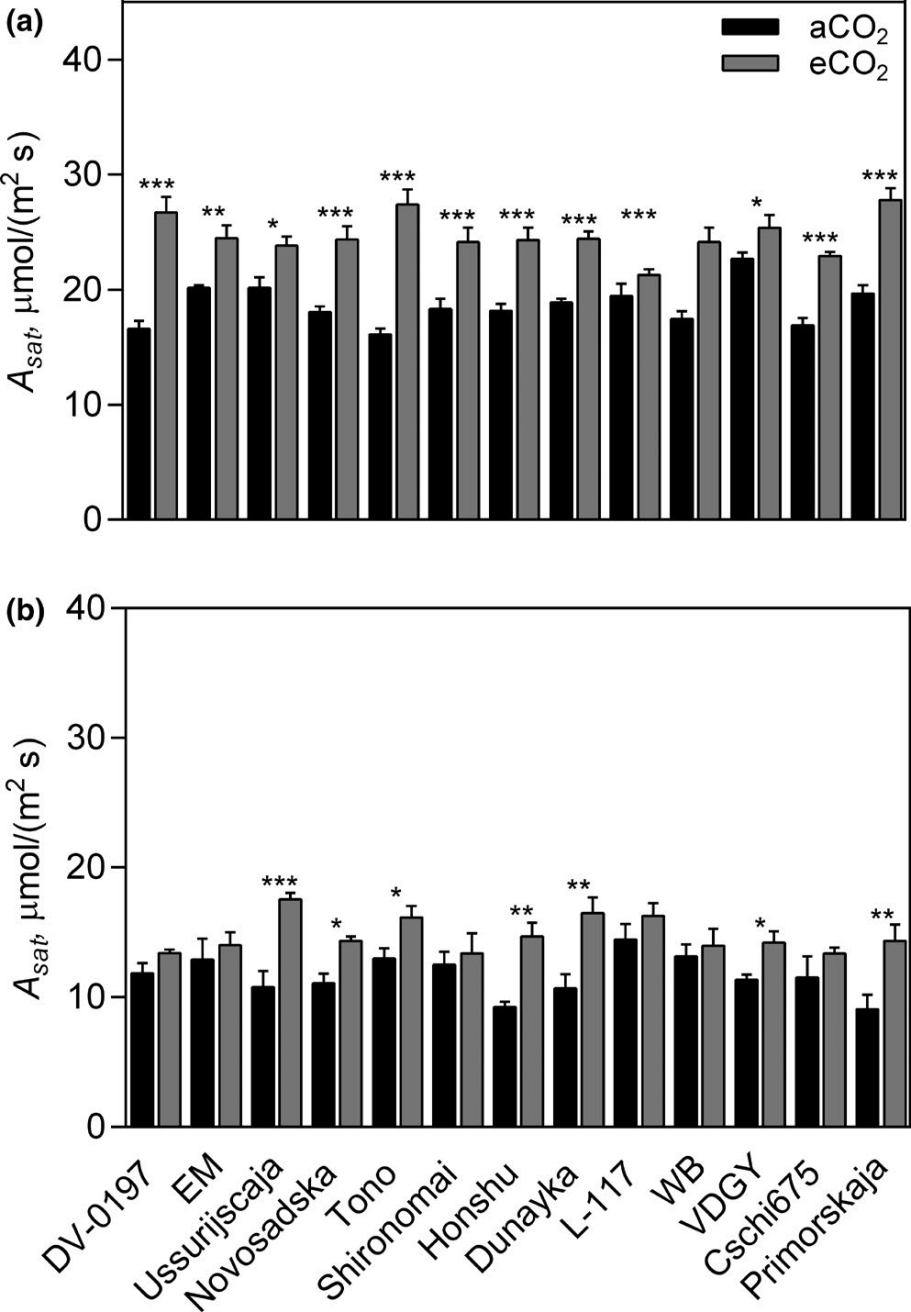
Photosynthetic CO_2_ assimilation of 13 soybean genotypes grown at aCO_2_ (400 ppm) and eCO_2_ (600 ppm). Values are the mean value ± standard error of the measurements made at (a) vegetative and (b) pod filling stages. Three plants from each subplot were sampled to assess the photosynthetic assimilation. * *p* < .05; ** *p* < .001; *** *p* < .0001

Growth at eCO_2_ significantly stimulated yield by 46.9% (*p* < .001; Figure 1a and Table 2) averaged across soybean genotypes under FACE conditions. The extent of yield improvement due to eCO_2_ differed significantly among the genotypes (*p* < .001), with a significant CO_2_ × genotype interaction (*p* < .01). The seed yield increase of Primorskaja (89.7%) was greatest, followed by Cschi675 (75.4%), VDGY (75.0%), and WB (55.7%), whereas in DV‐0197, EM, and Ussuriscaja, no stimulation in seed yield was observed. WB evidenced the greatest seed yield at both CO_2_ concentrations used in this study. Exposure to eCO_2_ slightly decreased the harvest index by 4.0% (*p* > .05), with a significant difference among genotypes (*p* < .05, Figure 2 and Table 2). Moreover, the genotypes with the highest decrease in harvest index were genotypes with no yield improvement.

**TABLE 2:** Analysis of variance of yield parameters in soybean genotypes exposed to aCO_2_ (400 ppm) and eCO_2_ (600 ppm), and correlations (Pearson's r) and their statistical significance for the relationship between the relative increase in yield due to eCO_2_ (value at eCO_2_/value at aCO_2_) and values of other parameters measured under the same conditions

Variables	CO_2_ effect	CO_2_			Genotype			CO_2_ × G			Correlation	*p*
	(%)	*F*	*P*	*df*	*F*	*p*	*df*	*F*	*p*	*df*		
Seed yield, g/plant	46.94	**181.88**	**<.001**	1	**21.40**	**<.001**	12	**3.33**	.**005**	12	−	−
No pods/plant	63.27	**297.48**	**<.001**	1	**16.85**	**<.001**	12	**3.43**	.**004**	**12**	**0.668**	.**013**
No of seeds/plant	60.25	**204.48**	.**001**	1	**12.70**	.**001**	12	**3.02**	.**009**	**12**	**0.865**	**<.001**
No of seeds/pod	−3.33	1.60	.218	1	**2.51**	.**024**	12	**3.68**	.**003**	12	0.492	.088
100‐seed weight, g	−11.86	**44.26**	.**001**	1	**35.15**	.**001**	12	**2.81**	.**013**	12	−0.082	.789
Harvest index, g/g	−4.03	1.61	.217	1	**4.01**	.**001**	12	1.13	.381	12	0.497	.084

Note

Results from the analysis of variance with degrees of freedom (*df*), F ratios and probabilities (*p*) for some plant parameters. Significant effects are shown in boldface.

ANOVA results showed that the aboveground dry weight was highly significant (*p* < .05) for CO_2_, growth stage, CO_2_ × genotype, CO_2_ × growth stage, genotypes ×growth stage, and interaction of CO_2_ × genotype ×growth stage (Table 4). Under eCO_2_ aboveground biomass was stimulated by 97.2% at the vegetative stage (*p* < .05, Figure 1c and Table 3) among soybean genotypes, while the increase in biomass was not statistically significant (*p* > .05) in Novosadska genotype. At the pod filling stage, eCO_2_ increased aboveground biomass by 61.2% (*p* < .05, Figure 1d and Table 3) averaged among soybeans. This increase was significant (*p* < .05) in 10 out of 13 genotypes. SPAD readings were highly significant (*p* < .01) for genotype, growth stage, CO_2_ × growth stage, CO_2_ × genotype, genotype ×growth stage, and interaction of CO_2_ × genotype ×growth stage. Exposure to eCO_2_ increased height by 11.1 and 23.9% at vegetative and pod filling stages, respectively, and there was a significant effect for CO_2_, genotype, growth stage, CO_2_ × growth stage, CO_2_ × genotype, genotype ×growth stage, and interaction of CO_2_ × genotypes ×growth stage. Moreover, leaf area increased by 88.5 and 59% at the vegetative and pod filling stages, respectively, due to the exposure to eCO_2_ conditions. There was a significant effect for CO_2_, growth stage, CO_2_ × growth stage, CO_2_ × genotype, genotype ×growth stage, and interaction of CO_2_ × genotypes ×growth stage (Table 4).

**TABLE 3:** Analysis of the response characteristics in soybean genotypes exposed to aCO_2_ (400 ppm) and eCO_2_ (600 ppm) at the vegetative and pod filling stages

Growth stage	[CO_2_]	ADW	SPAD	Height	Leaf area	*A*_sat_	*g*_s_	*T*_r_	*A*/*g*_s_	Fq΄/Fm΄
Vegetative	Ambient	1.45	30.89	15.39	182.28	18.67	0.30	4.99	62.02	0.38
	Elevated	2.87	33.09	17.09	343.67	24.69	0.28	4.97	86.18	0.36
	Mean change (%)	97.23	7.12	11.05	88.54	32.24	−6.67	−0.1	39.3	−5.26
Pod filling	Ambient	52.87	35.99	48.93	4234.08	11.66	0.10	2.28	123.50	0.24
	Elevated	85.21	34.33	60.63	6732.70	14.75	0.10	2.51	156.38	0.17
	Mean change (%)	61.17	−4.61	23.91	59.01	26.50	2.04	10.1	27.1	−29.17

Note

ADW, aboveground dry weight; Fq΄/Fm΄, photosynthetic light‐use efficiency. ADW (g/plant), height (cm), leaf area (cm^2^/plant), *A*_sat_ (µmol/(m^2^ s)), *g*_s_ (mol/(m^2^ s)), *T*_r_ (mol/(m^2^ s)), *A*_sat_ /*g*_s_ (µmol/mol).

**TABLE 4:** Analysis of variance of the response characteristics in soybean genotypes exposed to aCO_2_ (400 ppm) and eCO_2_ (600 ppm)

Effect	*df*	ADW	SPAD	Height	Leaf area	*A*_sat_	*g*_s_	*T*_r_	*A*/*g*_s_	Fq΄/Fm΄
CO_2_	1	**497.8, <.01**	3.57, .06	**364.29, <.01**	**206.26, <.01**	**317.0, <.01**	**7.97, <.01**	**8.87, <.01**	**475.7, <.01**	**49.42, <.01**
Genotype (G)	12	1.28, .25	**6.46, <.01**	**10.15, <.01**	1.69, 0.086	2.05, .023	**4.87, <.01**	**2.35, <.01**	**4.40, <.01**	**4.70, <.01**
Stage (S)	1	**6663.2,<.01**	**316.85, <.01**	**3065.94,<.01**	**4286.02,<.01**	**929.6, <.01**	**1396.3, <.01**	**1850.1, <.01**	**3298.8, <.01**	**97.32, <.01**
CO_2_ × G	12	**8.28, <.01**	**5.85, <.01**	**4.94, <.001**	**4.12, <.01**	**3.24, <.01**	**4.96, <.01**	**2.48, <.01**	**2.26, .011**	**3.09, <.01**
CO_2_ × S	1	**15.01, <.01**	**115.54, <.01**	**362.00, <.01**	**169.59, <.01**	**27.19, <.01**	**4.77, 0.03**	**8.45, <.01**	**339.5, .01**	**23.00, <.1**
G × S	12	**7.97, <.01**	**6.79, <.01**	**10.14, <.01**	**1.99, .036**	**3.22, <.01**	**4.97, <.01**	**2.53, <.01**	**4.16, .01**	**2.62, <.01**
CO_2_ × G × S	12	**3.93, <.01**	**4.21, <.01**	**4.94, <.01**	**3.42, <.01**	**2.95, <.01**	**4.13, <.01**	**2.48, <.01**	**2.31, <.01**	**4.19, <.01**

Note

ADW, aboveground dry weight; Fq΄/Fm΄, photosynthetic light‐use efficiency. ADW (g/plant), height (cm), leaf area (cm^2^/plant), *A*_sat_ (µmol/(m^2^ s)), *g*_s_ (mol/(m^2^ s)), *T*_r_ (mol/(m^2^ s)), *A*_sat_/*g*_s_ (µmol/mol). Results from the mixed model analysis of variance with degrees of freedom (*df*), F ratios and probabilities (*p*) for some plant parameters. Significant effects are shown in boldface.

The yield parameters including the number of pods per plant (mean CO_2_ effect of 63.3%, *p* < .001), number of seeds per plant (mean CO_2_ effect of 60.3%, *p* < .001), and 100 seed weight (mean CO_2_ effect of −11.9%, *p* < .001) were significantly affected by eCO_2_ conditions. However, the number of seeds per pod was not significantly (*p* > .05) changed by eCO_2_ conditions. ANOVA showed that these yield parameters were highly significant (*p* < .05) for genotype, and interaction of CO_2_ × genotype (Table 2).

### 3.2 Correlations between yield responses to eCO_2_

The relationships between the relative increase in grain yield at eCO_2_ (i.e. the value at eCO_2_/value at aCO_2_) were used to investigate how seed yield responses to eCO_2_ correlated with different variables affecting yield. Consequently, the number of pods per plant were positively and significantly correlated (*r *= 0.67, *p* < .05) with the magnitude of seed yield response to eCO_2_ (Table 2). The number of seeds per plant had also a strong positive correlation (*r *= 0.87, *p* < .001) with yield responses. These results indicate that genotypic variation in CO_2_‐based responses could be explained primarily by the higher pod production and consequently by the increased number of seeds per plant. Although no other parameters were significantly correlated with yield responsiveness to eCO_2_, the plasticity in pod production seems to play an essential role in soybean yield improvement.

### 3.3 Photosynthetic assimilation rate and gas exchange parameters

ANOVA results showed that gas exchange parameters (*A*_sat_, *g*_s_, *T*_r_, and *A*_sat_/*g*_s_) were significantly (*p* < .05) affected by CO_2_, genotype, growth stage, CO_2_ × genotype, CO_2_ × growth stage, genotype × growth stage, and interaction of CO_2_ × genotype × growth stage (Table 4). The average of *A*_sat_ across the soybean genotypes and the growing stages varied from 9.1 to 22.7 μmol/(m^2^ s) under aCO_2_ and from 13.4 to 27.8 μmol/(m^2^ s) under eCO_2_ (Figure 2). Elevated CO_2_ increased significantly (*p* < .05) *A*_sat_ in all genotypes, except for L‐117, at the vegetative stage, while this stimulation was only significant in seven genotypes at the pod filling stage (Figure 2a,b). When plants were at the vegetative stage, *g*_s_ decreased by 6.7% on average across genotypes, *A*_sat_/*g*_s_ increased by 39.3%, and *T*_r_ slightly decreased by 0.1%. At the pod filling stage, *g*_s_ increased by 2.0%, *T*_r_ by 10.1%, and *A*_sat_/*g*_s_ by 27.1% (Table 3).

### 3.4 Chlorophyll fluorescence transients

The photosynthetic light‐use efficiency (Fq΄/Fm΄) was investigated using the automated LIFT system. ANOVA results showed that Fq΄/Fm΄ was significantly (*p* < .01, Table 4) affected by CO_2_, genotype, growth stage, CO_2_ × genotype, CO_2_ × growth stage, genotype ×growth stage, and interaction of CO_2_ × genotype × growth stage. The Fq΄/Fm΄ values ranged from 0.28 to 0.44, and from 0.05 to 0.35 at the vegetative and pod filling stages, respectively (Additional file: Figure S1). When plants were at the vegetative stage, a significant decrease in Fq΄/Fm΄ was observed in EM, Ussurijscaja, Novosadska, and Tono. At the pod filling stage, the fluorescence measurements were delayed one week (late pod filling stage), regarding to the measurements of *A*_sat_, due to climatic conditions. Therefore, under eCO_2_ a decrease of 29.2% in Fq’/Fm’ values was observed (Table 3). This reduction was significant in EM, Tono, Shironomai, Honshu, WB, and L‐117 genotypes and was not changed in the remaining genotypes.

### 3.5 Grain nutritional analysis

**FIGURE 3:**
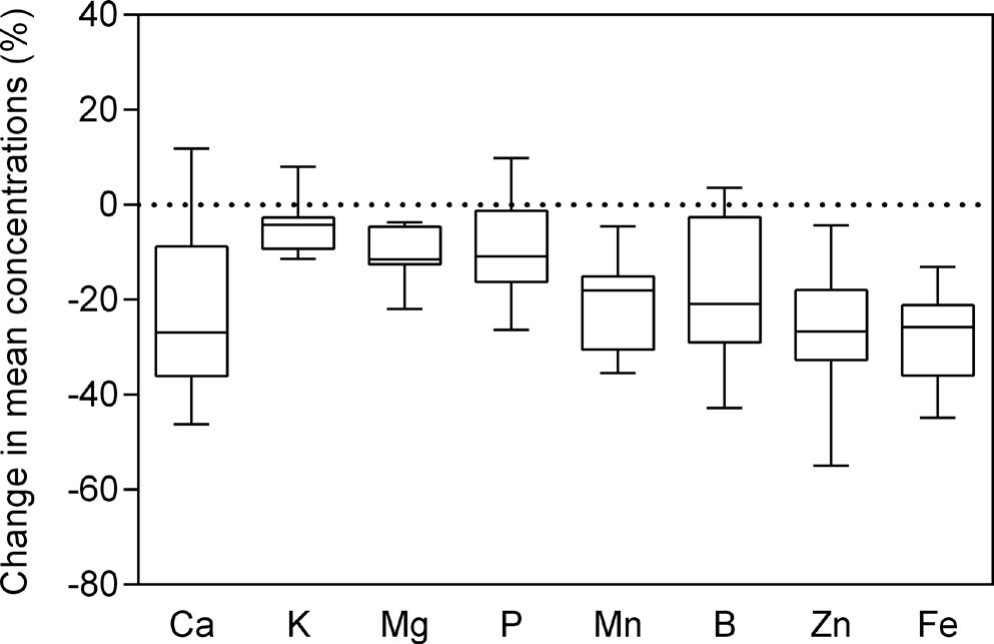
Boxplot shows the response ratio of the grain mineral concentrations of 13 soybean genotypes. CO_2_ response values are the mean value of each mineral at eCO_2_/aCO_2_. Ten seeds from independent plants from each subplot were pooled and used for mineral analysis

Elevated CO_2_ affected significantly mineral concentrations in soybean grains at maturity (Table 5). Calcium concentration decreased by 22.9% (*p* < .001, Figure 3 and Table 5) across soybean genotypes, and the concentrations responded differently to eCO_2_ among cultivars (*p* < .05), with a significant CO_2_ × genotype interaction (*p* < .001). The decrease was significant (*p* < .01) in EM, Honshu, Tono, Primorskaja, Dunayka, Cschi675, and Ussuriscaja (Table 6). Phosphorous (P) concentration was also reduced by 9.0% (*p* < .001), and changed significantly among genotypes (*p* < .001), with a significant CO_2_ × genotype interaction (*p* < .001). The concentration decreased by 15%, 26.3%, 20%, and 17.5% (*p* < .01) in Primorskaja, Cschi 675, Novosadska, and WB, respectively. Potassium concentration was reduced by 11.4%, 10.7%, and 9.5% in Cschi675, Novosadska, and WB, respectively. A reduction of 10.1% (*p* < .001) was observed in magnesium (Mg) concentration among all soybean genotypes. Therefore, it was observed a significant decrease by 12.8%, 21.9%, 12.5%, 11.4%, and 12.6% (*p* < .01) in Honshu, Cschi675, Novosadska, WB, and VDGY, respectively. In terms of micronutrients, the reduction was greatest for Fe and Zn, decreasing by 28.1%, and 25.9% averaged among genotypes, respectively. Manganese (Mn) concentration was also significantly reduced by 33.3%, 34.8%, 27.7%, 24.4%, 22.4%, 18.1%, 16.7%, and 11.7% in EM, Honshu, Tono, Primorskaja, Dunayka, Cschi 675, Novosadska, and WB, respectively. Consistent decreases in boron (B) concentration among genotypes were also found under eCO_2_, with a reduction of 20.9%, 29%, 42.8%, 22.3%, 28.9%, 28.5%, and 33.7% in DV‐0197, Honshu, Tono, Primorskaja, Cschi 675, Novosadska, and Ussuriscaja, respectively (*p* < .05). The magnitude of variation in micronutrient concentrations varied significantly among genotypes, with a significant CO_2_ × genotype interaction (*p* < .001), except for Fe. Genotypes with high mineral content at eCO_2_ might be a crucial trait for breeding programs. Consequently, EM exhibited simultaneously the highest concentration of B (22.6 μg/g), Fe (55.2 μg/g), and Mn (20.2 μg/g), and L‐117 exhibited the highest content of P (4.1 mg/g), Ca (1.5 mg/g), and Zn (34 μg/g).

**TABLE 5:** Analysis of variance and significance levels of main effects and interactions of CO_2_ and genotypes in mineral concentrations and phytochemical profiles from soybean genotypes exposed to aCO_2_ (400 ppm) and eCO_2_ (600 ppm)

Mineral	CO_2_ effect	CO_2_			G			CO_2_ × G		
	(%)	*F*	*p*	*df*	*F*	*p*	*df*	*F*	*p*	*df*	
Ca, (mg/g)	−22.93	**85.72**	**<.001**	1	**11.91**	**<.001**	12	**3.54**	**<.001**	12	
P, (mg/g)	−9.02	**35.57**	**<.001**	1	**4.84**	**<.001**	12	**3.84**	**<.001**	12	
K, (mg/g)	−4.86	**24.81**	**<.001**	1	1.83	.06	12	**1.96**	.**04**	12	
Mg, (mg/g)	−10.11	**64.30**	**<.001**	1	**5.63**	**<.001**	12	1.59	.11	12	
Mn, (µg/g)	−21.29	**183.95**	**<.001**	1	**11.72**	**<.001**	12	**3.66**	**<.001**	12	
Fe, (µg/g)	−28.13	**79.51**	**<.001**	1	**7.40**	**<.001**	12	1.35	.21	12	
B, (µg/g)	−18.53	**113.37**	**<.001**	1	**11.20**	**<.001**	12	**5.74**	**<.001**	12	
Zn, (µg/g)	−25.90	**175.93**	**<.001**	1	**4.05**	**<.001**	12	**4.11**	**<.001**	12	
TPC, mg gallic acid/g	−5.39	2.62	.11	1	**7.23**	**<.001**	12	**4.84**	**<.001**	12	
ABTS, mmol Trolox/g	−36.87	**414.20**	**<.001**	1	**12.48**	**<.001**	12	**10.36**	**<.001**	12	
Sugar, %	9.07	**5.94**	.**02**	1	**5.03**	**<.001**	12	**1.96**	.**04**	12	
Starch, %	16.00	**6.74**	.**02**	1	**12.17**	**<.001**	12	0.41	.95	12	
Protein, %	−5.63	**37.44**	**<.001**	1	**3.51**	**<.001**	12	1.90	.05	12	
Phytate, %	8.10	**21.49**	**<.001**	1	**2.17**	.**015**	12	**3.01**	**<.001**	12	

Note

Results from the analysis of variance with degrees of freedom (*df*), F ratios and probabilities (*p*) for some plant parameters. Significant effects are shown in boldface.

**TABLE 6:** Effects of eCO_2_ on soybean seed mineral concentrations from soybean genotypes exposed to aCO_2_ (400 ppm) and eCO_2_ (600 ppm)

Genotype	Ca (mg/g)		P (mg/g)		K (mg/g)		Mg (mg/g)		Mn (µg/g)		Fe (µg/g)		B (µg/g)		Zn (µg/g)	
	aCO_2_	eCO_2_	aCO_2_	eCO_2_	aCO_2_	eCO_2_	aCO_2_	eCO_2_	aCO_2_	eCO_2_	aCO_2_	eCO_2_	aCO_2_	eCO_2_	aCO_2_	eCO_2_
L‐117	1.71	1.53	3.70	4.05	18.43	17.70	2.20	2.11	22.50	19.05	52.97	40.58	15.75	15.05	35.50	33.97
EM	**2.10**	**1.30**	3.57	3.92	18.14	17.38	2.23	2.08	**30.27**	**20.18**	**74.34**	**55.15**	21.86	22.64	38.94	32.79
DV‐0197	1.25	1.17	4.16	3.75	17.07	16.76	2.21	1.96	19.48	18.60	50.19	40.80	**21.68**	**17.16**	34.84	27.86
Honshu	**1.97**	**1.29**	4.33	3.83	18.17	17.66	**2.43**	**2.12**	**25.82**	**16.83**	**67.01**	**36.94**	**21.07**	**14.97**	**39.67**	**29.08**
Tono	1.53	0.96	3.66	3.79	17.49	17.03	2.13	2.04	**22.91**	**16.56**	**51.97**	**34.48**	**19.73**	**11.29**	**38.97**	**27.86**
Primorskaja	1.77	1.29	**4.47**	**3.80**	18.59	17.62	2.33	2.13	**24.32**	**18.38**	**60.32**	**41.23**	**21.37**	**16.60**	**40.63**	**29.14**
Dunayka	**1.82**	**1.22**	3.65	3.31	18.52	17.31	1.96	1.89	**24.21**	**18.79**	41.48	32.02	20.46	17.13	**39.14**	**26.37**
Cschi675	**1.39**	**0.93**	**4.69**	**3.45**	**18.56**	**16.44**	**2.38**	**1.86**	**19.88**	**16.28**	**52.50**	**32.41**	**24.54**	**17.46**	**37.51**	**24.79**
Novosadska	1.34	1.25	**3.98**	**3.19**	**18.45**	**16.48**	**2.15**	**1.88**	**22.57**	**18.76**	42.61	32.90	**18.41**	**13.17**	**32.38**	**24.19**
Shironomai	0.85	0.95	3.54	3.32	18.62	16.95	1.98	1.88	17.87	14.75	36.49	31.72	20.08	19.94	**52.61**	**25.43**
WB	1.31	1.17	**3.98**	**3.29**	**18.30**	**16.56**	**2.10**	**1.86**	**22.07**	**19.51**	**41.11**	**33.12**	19.96	20.57	**32.63**	**25.44**
Ussurijscaja	**1.36**	**0.73**	3.67	3.16	17.14	18.52	2.06	1.72	20.64	13.34	**51.28**	**29.44**	**25.26**	**16.74**	36.04	23.30
VDGY	1.24	0.92	4.18	3.72	19.00	18.36	**2.15**	**1.88**	20.54	17.52	47.05	33.56	23.40	18.69	34.89	30.59

Note

Significant differences (*p* < .05) between aCO_2_ and eCO_2_ within a genotype are shown in boldface.

**FIGURE 4:**
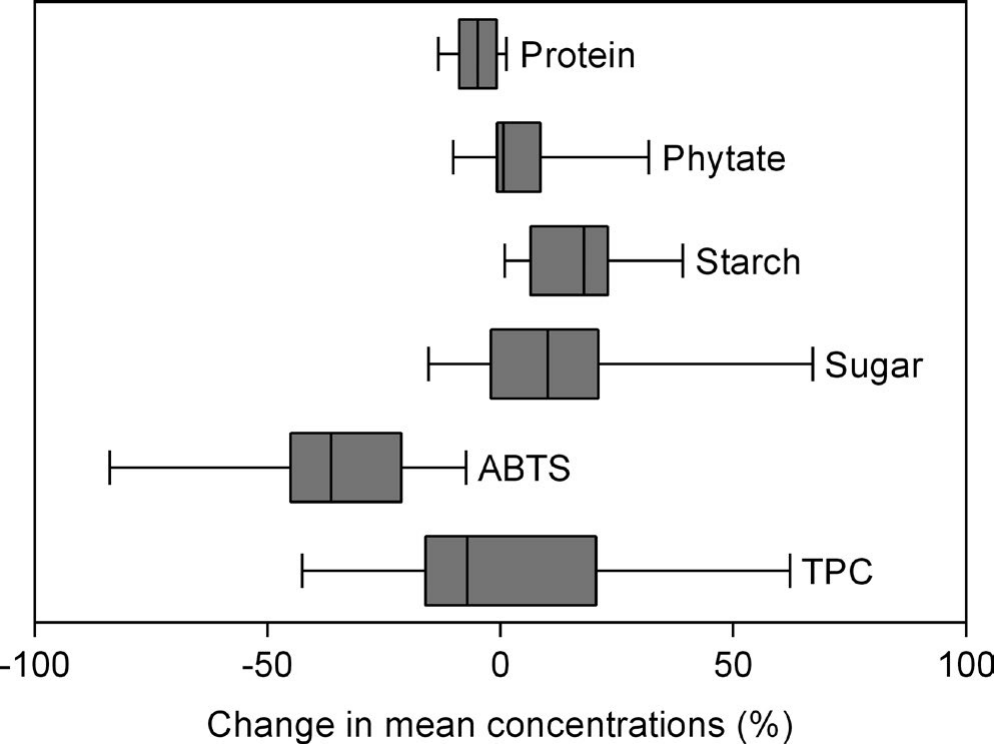
Boxplot shows the response ratio of the grain phytochemical profiles of 13 soybean genotypes under eCO_2_. CO_2_ response values are the mean value of each variable at eCO_2_/aCO_2_. Ten seeds from independent plants from each subplot were pooled and used for phytochemical analysis. TPC, total phenolic content; ABTS, 2,2′‐Azino‐bis (3‐ethylbenzothiazoline‐6‐sulfonic acid)

**FIGURE 5:**
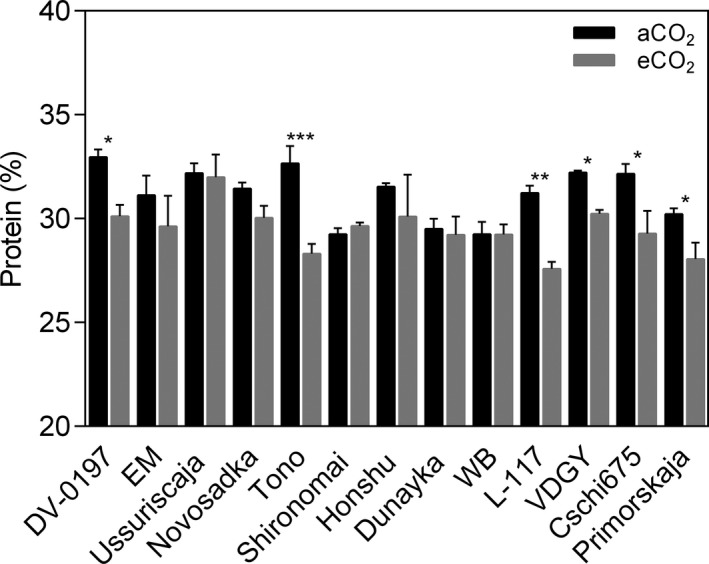
Genotypic variation in grain protein response under eCO_2_ conditions. Bars show the mean value ± standard error in 13 genotypes grown at the FACE facility in 2018. Ten seeds from independent plants from each subplot were pooled and used for protein analysis. * *p* < .05; ** *p* < .001; *** *p* < .0001

Elevated CO_2_ did not influence the TPC when compared with aCO_2_ (*p* > .05; Figure 4 and Table 5), but a significant difference across genotypes was observed (*p* < .001), with a CO_2_ × genotype interaction (*p* < .001). The ABTS values decreased significantly from 32.88 to 20.76 mmol Trolox/g (*p* < .001), with significant differences among genotypes (*p* < .001) and CO_2_ × genotype interaction (*p* < .001). Soluble sugar and starch concentrations in soybean grains improved due to eCO_2_ conditions by 9.1% and 16.0% (*p* < .05) averaged across soybean genotypes, respectively. We also evaluated phytate, a phosphate storage molecule that inhibits the absorption of some nutrients in humans. Phytate content increased significantly at eCO_2_ (*p* < .001), and the extent of change varied between genotypes (*p* < .05), with CO_2_ × genotype interaction (*p* < .001). Elevated CO_2_ reduced grain protein concentration by 5.6% (*p* < .001). This decrease was significant in Tono, L‐117, Cschi675, DV‐0197, Primorskaja, and VGDY with a reduction of 13.3, 11.7, 9.0, 8.6, 7.2, and 6.4%, respectively (Figure 5).

### 3.6 Nutritional analysis association with soybean yield

The PCA was performed in order to associate the responses at eCO_2_ of mineral concentrations and phytochemical profiles to that of grain yield. The results (additional file: Figure S2) shows the diversity of the samples, and also the identification of the variables responsible for that differentiation. The biplot revealed two principal components, together explaining 50.7% of the observed variability. The genotypes were mainly discriminated by PC1, with differentiation between genotypes growing at aCO_2_ and eCO_2_. The first principal component PC1 explained 38.1% of the variance showing a reduction in the grain nutritive value observed mainly through a decrease in mineral and protein concentrations, and also in the antioxidant activity. The genotypes positioned on the right‐hand side of the PCA plot were those grown under aCO_2_ conditions, and showed higher levels of the minerals, protein concentration, and antioxidant activity. The second principal component PC2 was responsible for 12.6% of the variation and positively correlated with starch and yield and negatively correlated with protein. Thus, PC2 showed the separation of the samples in the vertical direction, and the genotypes positioned in the higher half and on the left‐hand side of the PCA scores plot contained higher grain yield and starch content. Therefore, genotypes such as WB, Primorskaja, and L‐117 are probably good candidates for selection in future breeding programs mainly due to their high yield capacity and less affected in the grain quality. PCA shows that greater grain yield stimulation under eCO_2_ was associated with a reduction in mineral concentrations, probably suggesting a yield dilution effect.

## 4 DISCUSSION

Advances in soybean genetics, the discovery of new or improved genotypes, innovations in farming practices, and the increase in atmospheric CO_2_, have greatly contributed to increase in soybean yield. However, the extent of yield enhancement is possibly insufficient to meet the future demands of a growing global population (Bishop et al., 2015). This study showed genotypic variation in soybean yield responses under FACE conditions ranging from no significant changes, to an increase in seed yield of almost 90%, and the averaged increase was 46.9% among all genotypes (Figure 1 and Table 2). DV‐0197, EM, and Ussuriscaja did not increase seed yield under eCO_2_, whereas Primorskaja was the most responsive genotype to eCO_2_, followed by Cschi675, and VDGY. The best‐adapted genotypes to aCO_2_, were also the genotypes with the greatest seed yield at eCO_2_ (viz. WB, L‐117, and Shironomai) suggesting that the best‐adapted genotypes to the current CO_2_ might be useful in the upcoming CO_2_ concentration. The genotypes investigated in the current study were previously grown in a controlled environment (Table 1) under hydroponic conditions at aCO_2_ (400 ppm) and eCO_2_ (800 ppm) conditions (Soares, Deuchande, et al., 2019). The range of soybean yield responses to eCO_2_ was −23.8 to 39.6% with mean change of 7.1%. This contrasts with yield stimulation of 46.9% under FACE conditions corresponding to more than six times that of plants grown in hydroponic solutions. The reduction in seed yield increase was associated with the physical restriction in hydroponic root growth, since the volume for root growth was <2 L. Similarly, Ainsworth et al. (2002) also highlighted the effect of pot size in soybean growth and yield in a meta‐analysis. The authors described that even large pots (>9L) failed to predict the increase in yield seen in soybeans planted in the ground stimulation. Thus, seed yield increase in large pots was 12%, while yield stimulation of soybeans planted in the ground was 38% (Ainsworth et al., 2002). Therefore, there was little consistency between both studies. However, DV‐0197 and Ussuriscaja did not increase yield at eCO_2_ and were consistently unresponsive genotypes. Furthermore, WB and Shironomai showed a significant seed yield and biomass response to eCO_2_ under controlled environment and FACE conditions (Table 1). From our results, and others (Ainsworth et al., 2002; Bishop et al., 2015; Bunce, 2014; Kumagai et al., 2015; Soares, Deuchande, et al., 2019; Ziska et al., 2001) yield responses to CO_2_ enrichment varied considerably between genotypes, ranging from ‒10% to 90% for soybean (Bishop et al., 2015; Kumagai et al., 2015; Soares, Deuchande, et al., 2019; Ziska et al., 2001). Yield performance at eCO_2_ is essential for selecting CO_2_‐responsive genotypes. To our knowledge, only Bishop et al. (2015) described the genotypic variation in soybean responses under FACE conditions using more than two genotypes simultaneously. Furthermore, it was our purpose to understand which characteristics would best predict yield responses. We found that number of pods (*r *= 0.67, *p* < .05), and number of seeds per plant (*r *= 0.87, *p* < .001) were useful indicators of the yield responses at eCO_2_ conditions (Table 2). Moreover, the harvest index, that is, the proportion of biomass partitioned into seeds, was not significantly changed by eCO_2_ (*p* > .05). Therefore, in such conditions of more carbohydrates provided by photosynthesis stimulation, this suggests that there were no sink limitation restricting the capacity to generate more seeds. Therefore, the effect of CO_2_ was mainly an increase in biomass and, consequently, an increase in the number of pods that reached maturity with filled seeds. There was also a weak positive correlation between changes in harvest index (*r *= .497, *p* = .084, Table 2) and yield at eCO_2_, such that genotypes with significant reduction in harvest index showed no seed yield stimulation (viz. DV‐0197 and EM). We also analyzed photosynthetic parameters, and it was observed that yield prediction is not directly correlated from leaf photosynthesis due to the influence of other factors, such as respiration, leaf growth, partitioning of assimilates, flowering, and pod setting (Steduto et al., 1997). Our results demonstrated that eCO_2_ decreased leaf chlorophyll content (Table 3) at the pod filling stage implying that chlorophyll turnover might occur at this stage. It is generally accepted that photosynthesis acclimation occurs when the sink capacity is reduced (Morgan et al., 2001). In this study, we found an increase in *A*_sat_ under eCO_2_ at either vegetative and pod filling stages (Figure 2 and Table 3), as also a significant increase in pod formation to avoid sink limitation. Interestingly, Fq΄/Fm΄ values decreased at the pod filling stage and might be related to the start of leaf senescence and carbon remobilization to the new sinks. This could be explained by the fact that fluorescence measurements were made a week later than the gas exchange measurements due to the weather conditions.

We also studied the effects of CO_2_ concentration on the grain nutritional quality since CO_2_ enrichment can lead to changes in nutrients accumulation and pose a potential challenge to human health (Li et al., 2018). Data evaluation demonstrates that eCO_2_ shifts total mineral content toward a reduced level compared to aCO_2_; the mean change across all the minerals is ‒17.6%. Elevated CO_2_ significantly reduced Ca by 22.9%, P by 9.0%, K by 4.9%, Mg by 10.1%, Mn by 21.3%, Fe by 28.1%, B by 18.5%, and Zn by 25.9% (Figure 3). The magnitude of variation across mineral concentrations differed among soybean genotypes (*p* < .05), except for K (Table 5). The reduction in mineral concentrations was exacerbated under FACE conditions in relation to the growth chamber study of Soares, Deuchande, et al. (2019). This evidence probably reflects the significance of the greater dilution effect caused by the increase in carbon allocation in the current study. Loladze (2014) also found a decline in P, K, Ca, Mg, and Zn concentrations in foliar and edible tissues under FACE conditions, including wheat, barley, and rice. A reduction in grain Fe concentration has been reported in rice, wheat, barley, pea, and soybean, and Mn in rice and pea at FACE conditions (Myers et al., 2014). Wu et al. (2004) also suggested that nutrient concentrations (N, P, K, Zn) in wheat grains decreased by eCO_2_. This phenomenon increases the incidence of nutrient deficiency and other related diseases, and current plant breeding programs have been focused on higher yields instead of preserving grain nutritional quality (Fernando et al., 2014). Consequently, genotypes with high mineral content and high yield capacity under eCO_2_ might be important traits from a breeding perspective. Thus, among the high‐responsive genotypes, L‐117 had simultaneously the highest concentration of P, Ca, and Zn. The exact mechanisms for the decrease in grain mineral concentrations remain unclear. Some authors have proposed this phenomenon to the dilution effect caused by the increased biomass under eCO_2_ (Gifford et al., 2000; Li et al., 2019; Parvin et al., 2019). However, inhibition of photorespiration and malate production, decreased mass flow due to reduced transpiration rate might also be relevant in explaining the reduced mineral levels under eCO_2_ conditions (Bloom, 2015; Gifford et al., 2000; Pleijel et al., 2000). Legumes are a great source of phenolic compounds which play substantial roles in many physiological and metabolic processes, and are directly related to the antioxidant activity (Singh et al., 2017). Data obtained in this study showed that plants grown under eCO_2_ have lower antioxidant activity by 36.9%, but no significant effect was found on the TPC (Figure 4 and Table 5). These findings are consistent with previous studies showing that eCO_2_ could induce a decrease in antioxidant capacity in fruit vegetables (Dong et al., 2018), rice (Goufo et al., 2014), and soybean leaves (Gillespie et al., 2012). Pérez‐López et al. (2018) suggested that CO_2_ enrichment can reduce photorespiration, decreasing the formation of oxygen radicals, showing no need to induce antioxidant synthesis. This eCO_2_‐induced decrease in antioxidants of soybean seeds might have a great influence on human diet and on the food industry that produces antioxidants from soybean grains (Zheng et al., 2020). In the current study, eCO_2_ increased sugar, and starch in soybean grains by 9.1% (*p* < .05), and 16.0% (*p* < .05), respectively; whereas, mean values of seed protein was lowered by 5.6% (*p* < .001, Table 5). Besides, CO_2_ enrichment increased the concentration of soluble sugars in potato, and starch in potato and wheat using open‐top chambers as described by Högy and Fangmeier (2008) and Kumari and Agrawal (2014). Although soybean plants can symbiotically fix N, to alleviate N deficiency, shortcomings still occur under eCO_2_ conditions. Many studies support that lower seed protein concentration at eCO_2_ can be attributed to accumulation of non‐structural carbohydrates (Gifford et al., 2000; Wu et al., 2004). This evidence was supported by the greater increase in plant biomass, and consequently a great reduction in protein content, under FACE conditions as opposed to the growth chamber experiment described by Soares, Deuchande, et al. (2019). However, other mechanisms than carbohydrate dilution alone, might all be relevant to explain this phenomenon (Dietterich et al., 2015; Myers et al., 2014; Soares, Deuchande, et al., 2019). Thus, lower levels of protein could have nutritional implications for humans that use these crops as a food source. We also report phytic acid, a molecule present in most plants that has the potential for binding to positively charged protein, amino acids, and minerals in foods reducing their absorption in the human gut (Weaver & Kannan, 2002). This molecule increased at eCO_2_ by 8.10% (*p* < .01, Figure 4 and Table 5), and might intensify complications of nutrient deficiency. At eCO_2_, an increase of 1.2% and 12.8% in phytic acid concentration was also found in rice and sorghum, respectively (Myers et al., 2014). Therefore, genotypes such as WB, Primorskaja, and L‐117 are probably good candidates for selection in future breeding programs mainly because of their yield capacity and resilience to grain quality losses.

## 5 CONCLUSION

In conclusion, this study showed that there is a variation among soybean genotypes grown in field conditions under eCO_2_ conditions and that genetic background has the potential to adapt to the upcoming atmospheric CO_2_ concentrations. Exploiting this genetic diversity in crops can help to mitigate the negative impacts of climate change and improve crop yields in the future. Our results suggest that eCO_2_ has positive effects on the soybean yield but decreases the grain content of protein, minerals, and antioxidant capacity. However, it does appear that yield increase was driven by responsiveness in number of pods, and increased number of seeds. Therefore, it is essential to design strategies with a focus on increasing yield responses and select genotypes with minor nutritional losses that may occur under eCO_2_. Overall, WB, Primorskaja and L‐117 genotypes appear to be particularly promising to breed soybean to the future atmospheric conditions.

## CONFLICT OF INTEREST

The authors declare no conflicts of interest.

## AUTHOR CONTRIBUTION

José C. Soares, Lars Zimmermann, and Nicolas Zendonadi dos Santos performed the experiments, including the field experiments. José C. Soares, Onno Muller, and Marta W. Vasconcelos designed the project and experiments; José C. Soares wrote and edited the manuscript; Onno Muller, Manuela Pintado, and Marta W. Vasconcelos reviewed the manusccript. All authors read and approved the final manuscript.

## Supporting information

Fig S1

Fig S2

## Data Availability

The data that support the findings of this study are openly available in figshare at https://doi.org/10.6084/m9.figshare.16655071.
