# Supplementary figures and images for: Genotypic variation in the response of soybean to elevated CO2
Source: Plant Environ Interact. 2021 Dec 8;2(6):263–76. doi: 10.1002/pei3.10065 (PMC10168044; doi:10.1002/pei3.10065)

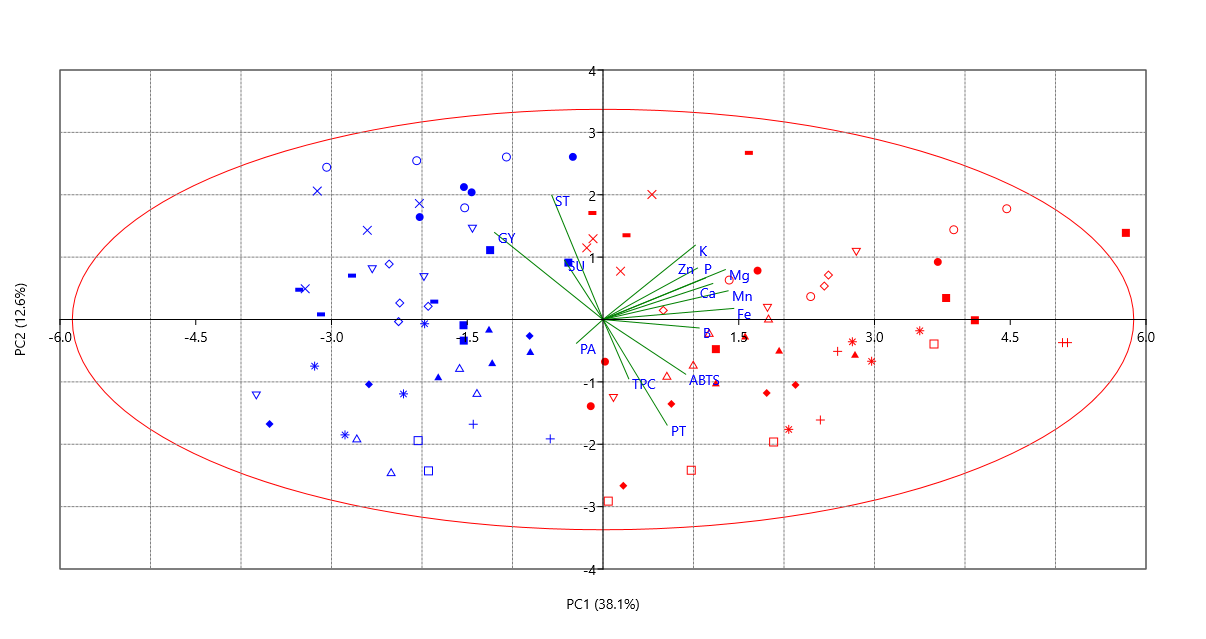

Supplement: Supplementary file 2 — Fig S2 [file PEI3-2-263-s002.tif]
